# Psychosocial impact and stigma on men who have sex with men due to monkeypox

**DOI:** 10.3389/fpubh.2025.1479680

**Published:** 2025-03-19

**Authors:** Rubén Linares-Navarro, Iván Sanz-Muñoz, Víctor Onecha-Vallejo, Virginia Fernández-Espinilla, Jose M. Eiros, Javier Castrodeza-Sanz, Camino Prada-García

**Affiliations:** ^1^Dermatology Service, Centro Sanitario Sandoval-Hospital Clinico San Carlos, Madrid, Spain; ^2^National Influenza Centre, Edificio Rondilla, Hospital Clínico Universitario de Valladolid, Valladolid, Spain; ^3^Instituto de Estudios de Ciencias de la Salud de Castilla y León, ICSCYL, Soria, Spain; ^4^Centro de Investigación Biomédica en Red de Enfermedades Infecciosas (CIBERINFECC), Madrid, Spain; ^5^Dermatology Service, Complejo Asistencial Universitario de León, León, Spain; ^6^Preventive Medicine and Public Health Service, Hospital Clínico Universitario de Valladolid, Valladolid, Spain; ^7^Microbiology Service, Hospital Universitario Río Hortega, Valladolid, Spain; ^8^Department of Preventive Medicine and Public Health, University of Valladolid, Valladolid, Spain

**Keywords:** mpox, monkeypox, MSM, homosexuality, anxiety, depression, stigma

## Abstract

**Background:**

The recent Monkeypox (Mpox) outbreak has disproportionately affected men who have sex with men (MSM), amplifying stigma and discrimination. While prior research examined media portrayals and public perceptions, little is known about MSM’s direct experiences. To address this gap, we assess discrimination, stigma, and psychosocial impact across social and healthcare settings.

**Methods:**

A cross-sectional observational study was conducted using a structured, pilot-tested survey to assess discrimination against MSM in media, family, socio-occupational, and healthcare environments.

**Results:**

Among 115 MSM surveyed, 81.7% observed discriminatory comments in media, while discrimination was noted in workplaces (41.7%), by family/friends (45.2%), cohabitants (15.7%), and healthcare (34.8%). Stigma significantly impacted healthcare-seeking behavior, with 33% avoiding medical care due to fear of discrimination. Psychologically, 50.4% reported low mood/anxiety, and 72.7% of those frequently fearing Mpox also experienced these symptoms. The outbreak led 71.3% to alter sexual behavior, primarily reducing encounters (60%). Fear of Mpox was strongly associated with behavioral changes (*p* < 0.001).

**Conclusion:**

The Mpox outbreak has exacerbated stigma toward MSM, highlighting an urgent need for intervention. Authorities, media, and community leaders must disseminate accurate information and implement psychological support programs to mitigate stigma and its detrimental effects on MSM.

## 1 Introduction

Monkeypox, now referred to as Mpox, is an infectious disease caused by the Mpox virus (MPXV), a double-stranded DNA virus belonging to the Orthopoxvirus genus, which includes the variola virus responsible for smallpox (1). The disease was first identified in 1970 in the Democratic Republic of Congo (DRC) and was initially considered a zoonotic infection with limited human-to-human transmission. Historically, Mpox was confined to rural, rainforest regions of central and western Africa, primarily transmitted to humans through direct contact with the blood, bodily fluids, or lesions of infected animals, particularly rodents and primates. However, in recent years, there has been an increase in Mpox cases outside Africa, raising concerns about its potential to become a global health threat. The emergence of Mpox outside endemic regions reflects broader changes in global health patterns influenced by factors such as increased human-animal interactions, climate change, and global travel (2–4).

In May 2022, cases of Mpox were reported in Europe, especially in Spain and the United Kingdom, and have since spread to other continents (2). These outbreaks in non-endemic countries have raised significant concerns among global health authorities about the potential for Mpox to establish new endemic regions, particularly in urban areas with dense populations and high levels of human interaction. On July 23, 2022, the World Health Organization (WHO) declared the escalating global Mpox outbreak a Public Health Emergency of International Concern (PHEIC) (5–8) and on May 11, 2023, WHO announced that Mpox was no longer a PHEIC considering a significant decline in reported cases, with no changes in disease severity or clinical manifestation (9, 10). The new Mpox outbreak has mainly affected men who have sex with men (MSM) (11–13). According to a Euro surveillance survey in Spain, 93% of Mpox patients, out of a total sample of 427 cases, identified as MSM (14). To address these outbreaks, countries applied preventive measures like active case finding, contact tracing, self-isolation, and quarantine (15). While effective in controlling the spread of the virus, these measures also contributed to significant social and psychological challenges for the MSM community. The association of the virus with this group has led to increased stigma and discrimination, exacerbating existing vulnerabilities and potentially leading to underreporting of cases due to fear of social repercussions (16). On November 28, 2022, WHO adopted “Mpox,” as a synonym for monkeypox to combat the spread of racist and stigmatizing language (17, 18).

As of 2024, the situation in Africa has worsened, particularly in the DRC, where the virus remains a major public health issue (19). WHO declared Mpox a global emergency for the second time on August 14, 2024, citing the alarming rise in cases caused by clade I of the virus, associated with a higher mortality rate compared to clade II variants that drove the 2022 global outbreak. This clade I variant, which includes the newly emerged subclade Ib, is now spreading in several African nations and has been reported outside the continent, raising concerns about its global impact (20). The reemergence of Mpox in new geographical and social contexts underscores the need for comprehensive public health strategies addressing both biological and social determinants of the disease, including the prevention of stigma and discrimination against affected populations.

Stigma is described as a dynamic process in which one person (the stigmatizer) devalues another person or group (the stigmatized). This concept highlights the social and psychological interactions that result in discrimination and marginalization (21). Stigma not only undermines individual well-being but also exacerbates health disparities by discouraging affected individuals from seeking healthcare services (22). This stigmatization process not only affects individuals psychologically but also poses significant public health risks by discouraging affected individuals from seeking medical care and adhering to preventive measures (23). The stigmatization seen during the Mpox outbreak is reminiscent of the early days of the Human Immunodeficiency Virus/ Acquired Immunodeficiency Syndrome (HIV/AIDS) epidemic, where misinformation and prejudice fueled widespread fear and discrimination, leading to substantial health disparities (22). This process leads to health inequality by creating imbalanced societal conditions that marginalize certain populations.

The WHO defines health-related stigma as a negative association between a group of people and a specific disease (24). Health-related stigma can manifest in various ways, including social exclusion, discrimination in healthcare settings, and internalized stigma among those affected. The impact of such stigma is profound, leading to delayed diagnoses, reduced access to healthcare, and poor mental health outcomes (25). However, despite these well-documented effects, there remains a gap in understanding how stigma is experienced in daily life by those affected. While previous studies have explored Mpox-related stigma, most have focused on media narratives (26), policy recommendations (27), and public health responses (28), rather than directly capturing the lived experiences of affected individuals. Research such as that of Zimmermann et al. (29) has analyzed anticipated stigma and social perceptions in the Netherlands, while Nerlich and Jaspal (26) examined how Mpox was framed in news media. However, empirical data on how stigma tangibly impacts healthcare-seeking behavior, mental health, and daily social interactions among MSM remain scarce. This study fills that gap by providing first-hand evidence from MSM affected by Mpox in Spain, assessing their experiences of discrimination in various settings (media, workplace, family, and healthcare). By focusing on direct testimonies, this research contributes to a more comprehensive understanding of the social and psychological consequences of Mpox-related stigma and its intersection with health-seeking behaviors.

Patients, suspected infected people, or specific groups with attributes associated with infectious diseases are prime targets for stigma (30). MSM and people with HIV often perceive and internalize significant stigma from family members, healthcare providers, and community members due to their sexual behaviors. The current Mpox outbreak affects both communities (22, 25), and the intersectionality of stigma related to sexual behavior, disease, and social identity creates complex challenges for affected individuals, leading to compounded health disparities and poorer health outcomes. Stigmatization represents a serious public health concern that, during infectious outbreaks, can lead to more new cases and exacerbate underlying health vulnerabilities (27, 31, 32). Additionally, psychological disorders, such as depression and anxiety, are common among infected patients (33).

Among MSM cases, a significant proportion (20–25%) presented atypical clinical manifestations of Mpox and co-infections with other sexually transmitted diseases, complicating diagnosis and management (34). The current epidemic differs from previous outbreaks in terms of sources of infection, routes of transmission, vulnerable populations, sexual identity, and risky behaviors (35). Early recognition of cases, identification of risk factors, contact tracing, and strengthened surveillance are fundamental to containing a Mpox outbreak (36). Current public health strategies must evolve to address the unique challenges posed by this outbreak, including culturally sensitive communication strategies, targeted interventions for high-risk populations, and the development of stigma-reduction programs. Healthcare professionals need to consider this disease when conferring a differential diagnosis especially in high-risk individuals (16).

The Mpox outbreak has primarily affected MSM due to closely connected sexual networks, leading to rapid spread. Addressing the risk of infection without stigmatizing individuals is crucial (11).

A recent study by Owhonda et al. (37) found high levels of community and self-stigmatization among MSM which may lead to an “iceberg phenomenon” where the true incidence of Mpox is underestimated due to underreporting. In emerging infectious diseases, social stigma has led labeling, stereotyping, discrimination, and loss of status, with various detrimental repercussions (38).

The psychosocial impact and stigma associated with infectious diseases can severely affect individuals, leading to mental health challenges, social exclusion, and reduced access to healthcare, particularly among marginalized groups. This impact is compounded by existing social inequalities, making it imperative for public health interventions to address both the biological and social determinants of health in response to the Mpox outbreak (39) MSM often face unique challenges due to societal attitudes and prejudices, especially when living with conditions such as Mpox, a viral disease that has recently seen an outbreak in this population.

The aim of this study was to assess the psychosocial impact, discrimination, and stigma associated with the Mpox outbreak among MSM, providing insights into areas for improvement. It also contributes to a broader understanding of the challenges faced by MSM during public health crises, highlighting the need for targeted interventions to reduce stigma and its harmful effects.

## 2 Materials and methods

### 2.1 Study design

An observational, cross-sectional study was conducted between July and December 2022 to evaluate the psychosocial impact and stigma experienced by MSM during Mpox outbreak. This survey was meticulously designed to comply with the General Data Protection Regulation (GDPR) guidelines, thereby ensuring the confidentiality and protection of respondents’ personal information.

### 2.2 Study sample and settings

The study sample was composed of MSM who voluntarily participated in an anonymous online survey. It was designed using “Google Forms ®,” a platform that ensures participant anonymity by no requiring any personal identification data. This approach was crucial in minimizing the risk of bias and encouraging honest responses, especially given the sensitive nature of the topics being addressed, such as stigma and discrimination.

The survey was distributed via social media platforms, focusing on Instagram®, between July and December 2022. To reach the target population effectively, Instagram Stories® were shared through various accounts that had agreed to collaborate with the study. These accounts were selected for their influence and connection with the MSM community, ensuring broader and more relevant reach. Although the survey was accessible to all viewers, it was explicitly specified that the survey was intended for MSM only. Clear instructions and disclaimers were provided to guide participants, ensuring that responses were primarily collected from the intended demographic. While the survey was disseminated nationally with the aim of reaching MSM across all autonomous communities in Spain, the anonymity of responses precluded the collection of data on the geographical origin of participants, which is acknowledged as a limitation of the study.

### 2.3 Survey instrument

The survey consisted of a structured questionnaire with 16 closed-ended questions designed to assess various aspects of stigma and discrimination experienced by MSM in different settings, including media, family, work, social environments, and healthcare. The questionnaire also included items related to changes in sexual behavior, mental health impacts such as anxiety and depression, and participants’ perceptions of available information on Mpox (Table 1).

**Table 1:** Demographic and psychosocial profiles of MSM: discrimination and stigma in the Mpox outbreak.

Survey questions	*N*	(%)
I have noticed discriminatory comments or attitudes toward MSM in the media.		
Yes	94	(81.7)
No	21	(18.3)
I have noticed discriminatory comments or attitudes toward MSM in my work environment.		
Yes	48	(41.7)
No	67	(58.3)
I have noticed discriminatory comments or attitudes toward MSM from family members or friends.		
Yes	52	(45.2)
No	63	(54.8)
I have noticed discriminatory comments or attitudes toward MSM from my cohabitants (flatmates, family members, etc.).		
Yes	18	(15.7)
No	97	(84.3)
I have noticed discriminatory comments or attitudes toward MSM in the health care system: health centre, hospital, etc.		
Yes	40	(34.8)
No	75	(65.2)
Stigma or discrimination against MSM has sometimes stopped me from seeking medical care.		
Yes	38	(33.0)
No	77	(67.0)
I am afraid of catching Mpox.		
Yes, frequently	44	(38.3)
Sometimes	44	(38.3)
No, never	27	(23.5)
I have felt low mood or anxious.		
Yes	58	(50.4)
No	57	(49.6)
I have decided to change my sexual habits (multiple choice).		
I have reduced my number of sexual encounters.	69	(60.0)
More frequent use of condoms.	20	(17.4)
I have sought medical attention for testing for sexually transmitted diseases.	18	(15.7)
I have stopped frequenting certain places.	33	(28.7)
I have changed my sexual habits in another way.	32	(27.8)
I have not changed my sexual habits.	33	(28.7)
I consider it necessary that measures are taken to prevent discrimination against MSM due to Mpox.		
Yes	103	(89.6)
No	12	(10.4)
As for the information on Mpox available to me:		
It is insufficient and I would like to receive more information	78	(67.8)
It is sufficient	37	(32.2)
My age is:		
Between 18 and 25 years old	16	(13.9)
Between 25 and 35 years old	38	(33.0)
Between 35 and 45 years old	37	(32.2)
Over 45 years old	24	(20.9)
I live:		
With my partner	39	(33.9)
With relatives	30	(26.1)
With flatmates	15	(13.0)
Only	31	(27.0)
I work as:		
Private sector worker	40	(34.8)
Public sector worker	41	(35.7)
Self-employed/entrepreneur	9	(7.8)
Retired	0	(0.0)
Unemployed	11	(9.6)
Student	14	(12.2)
The municipality where I live has:		
Less than 20,000 inhabitants	18	(15.7)
20,000–100,000 inhabitants	17	(14.8)
100,000–500,000 inhabitants	36	(31.3)
More than 500,000 inhabitants	44	(38.3)
As for my level of education:		
Primary and secondary education	4	(3.5)
Vocational training	20	(17.4)
University students	90	(78.3)
None	1	(0.9)

The questions were formulated to be clear and concise, reducing the risk of misinterpretation and ensuring that the data collected was reliable and valid. The questionnaire was pilot tested with a small sample of MSM before full deployment to identify and correct any issues related to question clarity or survey logic.

To assess the internal consistency of the survey instrument, Cronbach’s alpha was calculated for the items addressing stigma, discrimination, and psychosocial impacts. The result was an alpha value of 0.74, indicating satisfactory reliability. This supports the robustness of the questionnaire and its ability to measure the intended constructs.

It also contained the following item, which had to be compulsorily accepted in order to deliver the results: “*I am of legal age and consent to the publication of the survey data. No personal data will be provided at any time. This survey is intended for MSM only*.” In this way, informed consent was obtained, and it was ensured that all participants were MSM of legal age.

### 2.4 Ethical aspects

Data collection for this study began after the approval of the study by Clinical Research Ethics Committee of León (CEIm), which issued a favorable report on 26 July 2022 (Approval Code: 2285). This approval certifies compliance with the applicable Spanish legislation on protected personal and bioethical data. All participants were informed of the study’s objectives, the anonymous nature of their personal data and responses, and the revocability of their consent at any time during and after the survey.

### 2.5 Data analysis

Survey data were exported from Google Forms® to IBM SPSS Statistics for Windows, Version 26.0 (IBM Corp, Armonk, NY) for analysis. Descriptive statistics, including frequencies and percentages, were calculated for each survey item to provide a comprehensive overview of the participants’ responses.

To examine the relationships between demographic characteristics, experiences of discrimination, stigma, and changes in sexual behavior, Chi-Square tests were conducted. Statistical significance was set at a *p*-value of less than 0.05, indicating meaningful associations between the variables under study.

## 3 Results

A total of 115 MSM participated in the survey. The results provide a comprehensive overview of the discrimination, stigma, and psychosocial impacts experienced by the respondents in various settings during the Mpox outbreak. The descriptive analysis of the survey is shown in Table 1.

### 3.1 Discrimination in different environments

Discrimination was most frequently observed in media contexts, with 81.7% of respondents reporting discriminatory comments or attitudes toward MSM following the Mpox outbreak. Workplace environments also showed significant levels of discrimination, with 41.7% of participants noting discriminatory behaviors. Similarly, 45.2% of respondents reported experiencing discrimination from family members or friends. Although less prevalent, 15.7% observed discriminatory attitudes from cohabitants, such as flatmates or family members. Within healthcare settings, discrimination was reported by 34.8% of participants, highlighting the challenges faced by MSM in accessing equitable care. These findings underscore the multifaceted nature of discrimination encountered by MSM across diverse environments. The perception of discriminatory comments or attitudes toward MSM was significantly associated with the emotional state of participants. Those reporting low mood or anxiety were more likely to notice discrimination in the media (89.7% vs. 73.7%, *p* = 0.023), in the workplace (60.3% vs. 22.8%, *p* < 0.001), from family or friends (63.8% vs. 26.3%, *p* < 0.001), from cohabitants (29.3% vs. 1.8%, *p* < 0.001), and within the healthcare system (43.1% vs. 26.3%, *p* = 0.045) (Table 2).

**Table 2:** Analysis of psychosocial impact factors related to Mpox among MSM.

Psychosocial factors		*n*/Total (%)	*p*
Low mood or anxiety based on fear of contracting Mpox			
- Fear of contracting Mpox	Yes, frequently	32/44 (72.7%)	**<0.001**
	Sometimes	17/44 (38.6%)	
	No, never	9/27 (33.3%)	
Changes in sexual behavior based on low mood or anxiety			
- Low mood or anxiety	Yes	51/58 (87.93%)	**<0.001**
	No	34/57 (59.65%)	
Perception of discriminatory comments or attitudes toward MSM in media based on low mood or anxiety			
- Low mood or anxiety	Yes	35/58 (60.3%)	**<0.001**
	No	13/57 (22.8%)	
Perception of discriminatory comments or attitudes toward MSM from family members or friends based on low mood or anxiety			
- Low mood or anxiety	Yes	37/58 (63.8%)	**<0.001**
	No	15/57 (26.3%)	
Perception of discriminatory comments or attitudes toward MSM from cohabitants (flatmates, family members, etc.) based on low mood or anxiety			
- Low mood or anxiety	Yes	17/58 (29.3%)	**<0.001**
	No	1/57 (1.8%)	
Perception of discriminatory comments or attitudes toward MSM in the healthcare system based on low mood or anxiety			
- Low mood or anxiety	Yes	25/58 (43.1%)	**0.045**
	No	15/57 (26.3%)	
Changes in sexual behavior based on fear of contracting Mpox			
- Fear of contracting Mpox	Yes, frequently	40/44 (90.9%)	**<0.001**
	Sometimes	34/44 (77.3%)	
	No, never	11/27 (40.7%)	
Need for preventive measures against discrimination toward MSM due to Mpox based on perceived sufficiency of information on Mpox			
- The information on monkeypox available to me is sufficient	Yes	33/37 (89.2%)	**0.581**
	No	70/78 (89.7%)	

Bold values represent statistically significant values (*p* < 0.05).

### 3.2 Impact of stigma on healthcare seeking behavior

Stigma had a tangible impact on the willingness of MSM to seek medical care. Approximately 33% of respondents stated that stigma or discrimination sometimes deterred them from seeking medical attention. This reluctance to access healthcare could potentially contribute to delayed diagnosis and treatment, exacerbating health outcomes. These findings provide empirical evidence that stigma acts as a direct barrier to healthcare access, a phenomenon often suggested in qualitative and theoretical research but less frequently documented with primary data. Unlike prior studies that focused on anticipated stigma or media discourse, this study captures real-life avoidance behaviors in response to Mpox-related stigma, emphasizing the urgent need for stigma-reduction strategies within healthcare systems.

### 3.3 Psychological impact

The stigma associated with Mpox significantly impacted healthcare-seeking behavior among MSM. Approximately 33% of respondents indicated that stigma or discrimination had sometimes deterred them from seeking medical attention. This reluctance to access healthcare services underscores the pervasive influence of stigma, which can lead to delayed diagnoses and worsen health outcomes. The fear of being judged or discriminated against in healthcare settings emerges as a critical barrier, emphasizing the need for interventions to create inclusive and supportive medical environments. The data also revealed a statistically significant relationship between fear of contracting Mpox and experiencing low mood or anxiety. Specifically, 72.7% of those who frequently feared contracting Mpox reported feeling low mood or anxiety (*p* < 0.001) (Table 2).

### 3.4 Changes in sexual behavior

The survey revealed significant changes in sexual behavior among MSM due to the Mpox outbreak. A majority of respondents (71.3%) reported altering their sexual habits in response to the outbreak. The most common change was a reduction in the number of sexual encounters, reported by 60% of participants. Additionally, 28.7% of respondents stopped frequenting places associated with social or sexual activity. These results extend previous research on stigma and infectious disease outbreaks by demonstrating how stigma-related fear translates into tangible behavioral modifications. Unlike earlier studies that primarily analyzed stigma through qualitative interviews or media narratives, this study quantitatively measures the extent to which MSM have adapted their behaviors due to both perceived health risks and social discrimination. Some participants adopted preventive measures, with 17.4% reporting increased condom use and 15.7% seeking medical attention for testing for sexually transmitted infections (STIs). Furthermore, 27.8% of respondents made other unspecified changes to their sexual behavior, reflecting a wide range of adaptive responses to the outbreak.

There was a significant correlation between experiencing low mood or anxiety and changes in sexual behavior. Those who reported feeling low mood or anxiety were more likely to modify their sexual habits (87.9%) compared to those who did not (59.6%) (*p* < 0.001) (Table 2).

The Table 2 also shows that the fear of contracting Mpox is strongly associated with changes in sexual behavior. Among participants who frequently feared contracting Mpox, 90.9% reported changes in their sexual behavior, compared to 77.3% of those who sometimes feared it, and only 40.7% of those who never feared it (*p* < 0.001).

### 3.5 Perception of information availability

A large proportion of respondents (67.8%) felt that the information available about Mpox was insufficient, indicating a strong desire for more comprehensive and accessible information about the disease, its transmission, and its impact on MSM.

### 3.6 Need for preventive measures against discrimination

The survey also revealed a broad consensus on the need for preventive measures against discrimination. An overwhelming 89.6% of respondents believed that additional measures should be implemented to prevent discrimination against MSM in the context of Mpox. These findings emphasize the need for structural and policy-level interventions to combat Mpox-related stigma. While previous research has documented MSM stigma in healthcare and social settings, few studies have provided quantitative insights into the perceived urgency of preventive measures within this population. This study underscores that, regardless of information availability, there is a strong and widely held demand for action against discrimination.

Regarding the perception of the need for preventive measures against discrimination toward MSM due to Mpox, no significant differences were found based on the sufficiency of the available information about Mpox (89.2% with sufficient information vs. 89.7% with insufficient information, *p* = 0.581).

### 3.7 Statistical analysis of demographic differences

Further statistical analysis was conducted to explore demographic differences in the experiences of discrimination and stigma:

- There were no significant differences in the experience of discrimination based on the type of job or the size of the municipality where the respondents lived.
- No significant differences were found in the perception of insufficient Mpox information based on the level of education. However, given the high percentage of participants with university-level education, this sample may not fully represent the general population.

## 4 Discussion

The findings of this study provide a comprehensive insight into the multifaceted stigma and psychosocial impact experienced by MSM during the 2022 Mpox outbreak. For members of the LGBTQI+ community, the stigma caused by a disease can be even greater due to the intersecting or layered stigma effect. Venereal and skin diseases are also a greater source of stigma than other pathologies. All these factors make MSM particularly susceptible to discrimination due to Mpox (31, 40–42) The results underscore the pervasive nature of discrimination in various environments, highlighting significant challenges in both public and private spheres that contribute to the marginalization of this community.

### 4.1 Discrimination across different environments

The high incidence of discrimination reported by participants, particularly in the media (81.7%), reveals the critical role that public discourse plays in shaping societal attitudes toward marginalized groups. The portrayal of Mpox in the media, often sensationalized, has likely reinforced harmful stereotypes, leading to widespread stigma against MSM (43). This media-driven stigma is not isolated; it permeates into other areas of life, as evidenced by the significant levels of discrimination reported in workplaces (41.7%), among family and friends (45.2%), and within healthcare settings (34.8%). The comparatively lower but still notable level of discrimination observed from cohabitants (15.7%) indicates that stigma penetrates even the most personal spaces of MSM individuals, affecting their day-to-day interactions and overall well-being (44).

These findings align with the broader literature on health-related stigma, which emphasizes how stigma is perpetuated across various social contexts and contributes to the social exclusion of affected individuals (45, 46). The pervasive nature of discrimination against MSM, particularly in times of public health crises, mirrors the stigmatization experienced during the HIV/AIDS epidemic. This historical parallel suggests that without targeted interventions, the stigma surrounding Mpox may become deeply entrenched, leading to long-term negative consequences for the MSM community (36). Yang et al. (22), based on the experience of the past thirty years of combating HIV-related stigma, have proposed a series of measures applicable to the current mpox outbreak. These measures focus on the three phases of the stigma formation process: emergence, development and proliferation.

The perception of discrimination in multiple contexts—ranging from media and workplaces to family and cohabitation settings—was significantly correlated with negative emotional states (Table 2). These findings underscore how discrimination not only serves as an external barrier but is also internalized, contributing to a significant psychosocial burden on MSM. It is crucial that interventions focus on reducing stigma in these environments, not only to improve public attitudes toward MSM but also to mitigate the negative effects on the mental health of this population.

### 4.2 Impact of stigma on healthcare seeking behavior

The significant impact of stigma on healthcare-seeking behavior is perhaps one of the most concerning findings of this study. It is well known that stigmatization is a barrier to seeking healthcare (47). The fact that 33% of respondents reported avoiding medical care due to fear of stigma underscores the profound barriers that discrimination creates in accessing essential healthcare services. This reluctance to seek care is not only detrimental to individual health outcomes but also poses a broader public health risk, as it may lead to delayed diagnoses and treatment, facilitating the continued spread of Mpox (48, 49). Addressing these challenges requires concerted efforts to combat stigma and ensure timely access to care (26, 50).

The avoidance of healthcare due to stigma is a well-documented phenomenon in the context of other infectious diseases, such as HIV (50, 51). The fear of being judged, discriminated against, or even outed in healthcare settings can be overwhelming, leading individuals to forego necessary medical interventions. Addressing this issue requires a concerted effort to foster a more inclusive and non-judgmental healthcare environment. Training healthcare professionals in cultural competence and sensitivity toward sexual health issues is critical to dismantling these barriers and ensuring that MSM feel safe and supported when seeking care. The findings of this study complement and expand on previous research by providing empirical evidence of how Mpox-related stigma directly affects MSM. Unlike studies that focus on public perceptions (29), media representation (26), or policy-level discussions (27), this research captures the tangible effects of stigma on healthcare avoidance, mental health deterioration, and social discrimination. Notably, the high percentage of MSM who reported modifying their sexual behavior or delaying medical consultations due to fear of judgment reinforces the urgent need for stigma-reduction strategies at both clinical and societal levels. These insights bridge the gap between theoretical discussions on stigma and its real-world consequences, underscoring the importance of integrating mental health support and stigma mitigation into public health responses.

### 4.3 Psychological impact and changes in sexual behavior

The psychological toll of the Mpox outbreak on MSM is evident in the significant proportion of respondents reporting low mood or anxiety (50.4%) and many likely experiencing depression as well. Depression is a critical aspect of mental health in this population, as it exacerbates vulnerabilities and reinforces stigma, which can further discourage healthcare-seeking behaviors. The association between the fear of contracting Mpox and psychological distress is particularly striking, with 72.72% of those frequently fearing infection also experiencing low mood or anxiety. This correlation highlights the mental health burden that public health crises can impose on already marginalized communities.

Moreover, the outbreak has led to notable changes in sexual behavior among MSM, with 71.3% of respondents altering their habits. The most common changes, such as reducing the number of sexual encounters (60%) and avoiding certain places (28.7%), reflect a heightened sense of caution driven by fear, anxiety and possibly depression (52). Additionally, the chemsex phenomenon may have played a role in amplifying the psychological impact of the outbreak. Previous studies suggest that chemsex is associated with risky sexual behaviors, increased substance use, and heightened susceptibility to mental health challenges, which may have further compounded the distress experienced by MSM during this public health crisis.

The significant correlation between low mood or anxiety and changes in sexual behavior (87.93% vs. 59.65%) suggests that mental health interventions are crucial in managing the broader impact of Mpox. Psychological support services, including counseling and peer support networks, could play a vital role in helping MSM navigate the complex emotions and behavioral adjustments prompted by the outbreak. By addressing the mental health needs of this community, we can mitigate the long-term psychosocial consequences of the epidemic (33).

### 4.4 Perception of information availability and the need for preventive measures

The overwhelming sentiment among respondents that the information available about Mpox was insufficient (67.8%) points to a significant gap in public health communication. This lack of accessible, comprehensive information may contribute to the spread of misinformation and exacerbate fears, ultimately reinforcing stigma. Effective communication strategies are essential in bridging this gap, ensuring that MSM receive accurate and timely information about the disease, its transmission, and preventive measures (53).

A number of studies have evaluated the content about mpox in different social networks such as Twitter®, Tik Tok® or YouTube® (38, 39, 54). Their analysis revealed that these media could be interpreted as a double-edged sword, capable both of spreading misinformation and stigma and of bringing positive messages to a large number of viewers (55, 56).

The lack of association between the perception of the sufficiency of available information about Mpox and the perception of the need for preventive measures against discrimination may indicate that, regardless of the available information, there is a strong consensus on the need to address discrimination. This suggests that educational efforts must be accompanied by concrete actions to prevent discrimination and cannot rely solely on improving information dissemination (Table 2).

Furthermore, the strong consensus on the need for additional preventive measures against discrimination (89.6%) underscores the urgent need for targeted interventions. Public health campaigns must prioritize stigma reduction, focusing on educating the broader public about Mpox in a way that avoids scapegoating specific communities. Collaborations with media outlets and social media platforms are particularly important to ensure that messaging is both accurate and non-stigmatizing.

### 4.5 Implications of demographic differences and study limitations

The lack of significant demographic differences in the experience of discrimination and stigma suggests that these issues are pervasive across various segments of the MSM community, regardless of factors such as job type or municipality size. However, the high percentage of participants with university-level education indicates that our sample may not fully represent the general population of MSM, potentially limiting the generalizability of the findings. Future research should aim to include a more diverse sample to better understand how different demographic factors might influence experiences of stigma and discrimination.

Additionally, the use of a cross-sectional design limits our ability to draw causal inferences from the data. While the associations observed are compelling, longitudinal studies are needed to establish causal relationships and track changes over time. The reliance on self-reported data also introduces potential biases, such as recall bias and social desirability bias, which may affect the accuracy of the findings. Despite these limitations, the study provides valuable insights into the impact of the Mpox outbreak on MSM and highlights the urgent need for interventions to address the associated stigma and discrimination. One of the strengths of this study is the anonymity of the survey, which likely increased participants’ willingness to disclose their experiences and perceptions regarding Mpox. This methodological approach minimized the risk of social desirability bias and ensured that sensitive topics, such as stigma and discrimination, could be explored with greater candor. Furthermore, the internal consistency of the survey instrument was evaluated using Cronbach’s alpha, which yielded a value of 0.74. This satisfactory level of reliability supports the robustness of the survey design and its ability to measure the intended constructs effectively.

## 5 Conclusion

The findings underscore the pervasive impact of stigma on MSM during the Mpox outbreak, highlighting how stigma permeates various aspects of life, from healthcare access to social interactions. Despite these challenges, the study identifies significant opportunities to mitigate the effects of stigma and prevent further harm. Public health authorities, media, and community leaders must take proactive measures to counteract MSM stigmatization. This includes the dissemination of accurate, evidence-based information through public health campaigns aimed at educating the general public and addressing misconceptions that fuel stigma. These efforts are crucial for promoting understanding and reducing fear within society.

Additionally, providing comprehensive psychological support to individuals affected by stigma is essential for managing the mental health impacts and building resilience within the MSM community against future public health crises. Healthcare systems, conventional media, and social media platforms have vital roles in fostering inclusive and supportive environments. Implementing these measures will yield benefits beyond the current Mpox outbreak, creating a framework for addressing stigma in future public health emergencies. Ultimately, the lessons learned from this outbreak can serve as a catalyst for lasting positive change in public health communication, societal attitudes, and the treatment of marginalized communities. Unlike previous research focused on media representations, this study highlights the direct consequences of stigma on healthcare access and behavioral changes among MSM. These findings provide empirical evidence of stigma-related healthcare avoidance, reinforcing the need for targeted interventions to mitigate stigma and its effects on public health.

## Data Availability

The raw data supporting the conclusions of this article will be made available by the authors, without undue reservation.
